# Envisioning a learning surveillance system for tuberculosis

**DOI:** 10.1371/journal.pone.0243610

**Published:** 2020-12-14

**Authors:** Suman Gadicherla, Lalitha Krishnappa, Bindu Madhuri, Susanna G. Mitra, Arkalgud Ramaprasad, Raja Seevan, S. D. Sreeganga, Nibras K. Thodika, Salu Mathew, Vani Suresh

**Affiliations:** 1 Department of Community Medicine, M S Ramaiah Medical College, Bengaluru, Karnataka, India; 2 Indian Centre for Social Transformation (Indian CST), Bengaluru, Karnataka, India; 3 Ramaiah Public Policy Center, Bengaluru, Karnataka, India; 4 Professor Emeritus of Information and Decision Sciences, University of Illinois at Chicago, Chicago, Illinois, United States of America; University of Ghana College of Health Sciences, GHANA

## Abstract

Surveillance is critical for interrupting transmission of global epidemics. Research has highlighted gaps in the surveillance for tuberculosis that range from failure to collect real-time data to lack of standardization of data for informed decision-making at different levels of the health system. Our research aims to advance conceptual and methodological foundations for the development of a learning surveillance system for Tuberculosis, that involves systematic collection, analysis, interpretation, and feedback of outcome-specific data. It would concurrently involve the health care delivery system, public health laboratory, and epidemiologists. For our study, we systemically framed the cyber environment of TB surveillance as an ontology of the learning surveillance system. We validated the ontology by binary coding of dimensions and elements of the ontology with the metadata from an existing surveillance platform—GPMS TB Transportal. Results show GPMS TB Transportal collects a critical range of data for active case investigation and presumptive case screening for identifying and detecting confirmed TB cases. It is therefore targeted at assisting the Active Case Finding program. Building on the results, we demonstrate enhanced surveillance strategies for GPMS that are enumerated as pathways in the ontology. Our analysis reveals the scope for embedding learning surveillance pathways for digital applications in Direct Benefit Transfer, and Drug Resistance Treatment in National TB Elimination Programme in India. We discuss the possibilities of developing the transportal into a multi-level computer-aided decision support system for TB, using the innumerable pathways encapsulated in the ontology.

## Introduction

Disease surveillance and response systems remain central to modern public health practices. The recent surges in epidemics underscore the critical role of surveillance systems in providing timely and reliable health information to inform operational and strategic decision-making for multi-level health systems. Current outbreaks of epidemics have exposed issues that range from gaps related to the collection of real-time data to the standardization of data to inform and improve decision-making at different levels of the health system [1]. Mounting evidence from countries with high TB and malaria cases indicate the risk posed by the loss of data and unresponsive surveillance system in the context of vastly heterogeneous high-burden settings [2]. A recent scoping review on digital interventions for TB highlights the lack of focus on health systems and resource management [3].

Our research introduces a promising first step to envision a learning surveillance system for TB. A learning surveillance system involves ‘repeated cycles of observation and data analysis to identify opportunities for improvement, and implementation of changes’ as a means ‘for rapidly converting data into actionable information to improve population health’ [4]. Its attributes are in line with the Learning Health Systems described as ‘cyber-social ecosystem’, to solve complex interdisciplinary problems of timely evidence, and to support best care practices [5]. As an emergent area of research, the concept has been applied to malaria elimination [6] and vector-borne diseases [7].

In this paper, we advance the conceptual and methodological foundations for the development of a learning surveillance system for TB. We present it as an ‘ontology’, described as a multidimensional cognitive framing that helps visualize the system and its pathways in structured natural English and serves as a roadmap for the design, development, and use of learning surveillance systems for TB management. We validate the framework by mapping and coding of the metadata from a surveillance platform—GPMS TB Transportal [8] onto the dimensions and elements of the ontology. Building on the results of the validation, we demonstrate surveillance strategies enumerated as dimensions, elements, and pathways in the ontology and discuss surveillance strategies in the light of India’s programmatic interventions for the National TB Elimination Programme (NTEP). Our analysis reveals the scope for embedding learning surveillance pathways through digital applications in Direct Benefit Transfer and Drug Resistance Treatment in National TB Elimination Programme in India.

## Methods and materials

### Ontology

We present the ontology as a cognitive map of the cyberenvironment for surveillance and monitoring of TB. Similar ontology has been applied to the study of healthcare [9], mHealth [10], national health policies [11], and other domains.

The ontology was developed in two phases from that of an earlier work on cyberenvironment for malaria surveillance proposed by two authors–one from computer science working on malaria surveillance, and the other from management information systems and strategic management [12] incorporating relevant elements that would capture the complex and dynamic landscape of surveillance presented in a conference. That ontology was first refined and extended for a project on developing a learning surveillance system for malaria elimination in India in collaboration with community medicine, public health, and healthcare systems experts [6]. The stakeholder dimension was added to the ontology in this phase. The modified malaria surveillance ontology was next adapted for TB surveillance in the present paper with multiple experts from the same disciplines who have been working on TB surveillance for a long time.

In the two phases, the dimensions and elements of the ontology were systematically synthesized from the taxonomies and terminologies of the following domains: management information systems, computer science, community medicine, public health, healthcare systems, and strategic management, as applied to malaria and TB surveillance respectively. The research and practice experts from these domains collaborated to systematize and mirror their personal and domain knowledge of the logic of TB surveillance in the ontology. The present ontology is a consensus of the collaboration.

The Timing and Surveillance dimensions are primarily based on information systems, epidemiology, and disease surveillance disciplines. Data and TB management are based on epidemiology, disease surveillance, community medicine, and public health disciplines. The Stakeholder dimension is based on strategic management, health systems, and public health disciplines.

Each phase of the ontology’s development was iterative. There were multiple iterations in each until a consensus on the logic was achieved among the diverse domain experts in the team. During each iteration, the team decided on the elements to be (a) included, (b) excluded, (c) combined–if they were synonyms, (d) subordinated–if they were hyponyms, and (e) superordinated–if they were hypernyms. Two tests of convergence to a consensus on the elements of a dimension were whether they were reasonably (a) mutually exclusive, and (b) exhaustive for TB surveillance, based on the research available and practice knowledge of the collaborators. The initial and subsequent ontologies have been validated by peer review and presentation at academic conferences [6, 7].

Almost all the collaborators were new to this method of visualizing the core logic of a problem using the structured natural-English of ontology. They understood and assimilated it in the process of development of the malaria and TB surveillance ontologies. At the time of the consensus, they perceived the logic of TB surveillance and its ontology to be isomorphic.

### Dimensions, elements, and pathways

**Fig 1:**
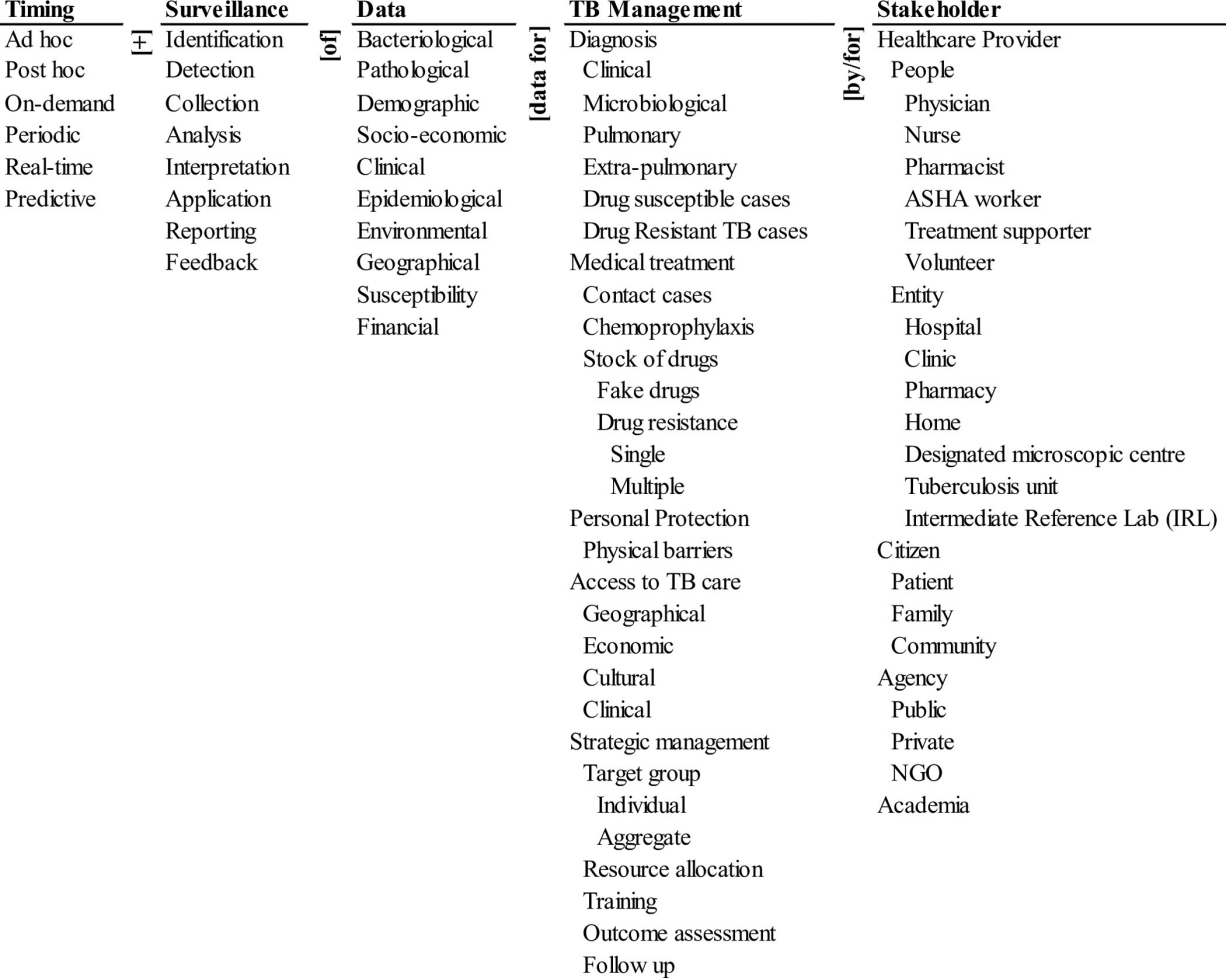
Ontology of a cyberenvironment for TB surveillance in India.

The ontology of a learning surveillance system for TB management in India is shown in Fig 1.

The main dimensions in the cognitive framing of the domain are identified as temporality, surveillance mechanisms, data, management, and stakeholders and represented as:

LearningSurveillanceSystemforTB=f(Timing+Surveillance+Data+TBManagement+Stakeholder)

Each of the dimensions is shown as a column and defined by a taxonomy of elements (Fig 1). *Data* describes programmatic data with regards to TB disease epidemiology, geographic spread, and demographic characteristics [13] along with socioeconomic, clinical, laboratory, and radiological, data from patient cases [14].

Data⊂[Bacteriological,Pathological,Demographic,Socio−economic,Clinical,Epidemiological,Environmental,Geographical,Susceptibility,Financial]

*TB management* describes information on clinical management of TB disease and access to TB services and focuses on managing symptoms, causes, treatment, and prevention of the diseases [15].

TBManagement⊂[Diagnosis,MedicalTreatment,Personalprotection,AccesstoTBCare,StrategicManagement]

*Stakeholders* involved are from the healthcare sector, external entities, and individuals engaged in the TB control ecosystem. These describe the important aspects related to public health accountability of TB for different stakeholders including the Government, healthcare functionaries, and citizens

Stakeholders⊂[Healthcareprovider,Entity,Citizen,Agency]

Combining these three columns enumerate various types of data used for managing the disease by different stakeholders. The three dimensions arranged together left to right with connecting terms (words and phrases) form natural English phrases that represent potential components of a simple information service to complex interaction services and hence, represent the information requirements of the surveillance system. For example, clinical data for active TB cases by a physician; or financial data for the stock of drugs by the hospital.

The three dimensions of the ontology are further concatenated with surveillance and timing that characterizes the tools and strategies for a learning surveillance system. Surveillance encapsulates case identification, detection, and data collection that are direct and cover both active and passive TB case finding at health facilities, communities during an investigation, and feedback activities. Timing represents the temporal dimension of the iterative process that determines the responsiveness of the system. It ranges from ad-hoc through real-time to predictive.

Surveillance⊂[Identification,Detection,Collection,Analysis,Interpretation,Application,Reporting,Feedback]

Timing⊂[Ad−hoc,Post−hoc,On−demand,Periodic,Real−time,Predictive]

The five dimensions are arranged left to right with connecting terms (rotated 90° clockwise) to form English sentences that represent the potential pathways for TB Surveillance. Each concatenation of a word/phrase from each column with the connecting words/phrases is a unique descriptor of a pathway for the learning surveillance system and represents a function of the system. Three illustrative pathways are:

- Real-time detection and collection of clinical and pathological data for source control by a public agency.
- Periodic collection and analysis of epidemiological data for drug-resistant cases by academicians.
- Predictive analysis of epidemiological data for transmission control by physicians.

All the pathways may not be instantiated in a system. Many may not be instantiable.

### Validation of the ontology

We validate the ontology by applying it to the analysis of an existing cyberenvironment for TB surveillance. The GPMS TB Transportal is a cloud-based platform that facilitates project/program/portfolio monitoring and evaluation with active crowdsourcing and citizen engagement [8]. It is an integrated dashboard that provides real-time data capture at source and aggregation at district and state levels. Its sources include a) India’s census data, b) data from NIKSHAY, a unified ICT system for TB patient management and care in India [16], and national health data on TB patients. It is also integrated into the Nikshay platform to which it feeds the case finding data. It also has facilities for direct patient contact through volunteers to healthcare providers to find missing and suspected cases [17].

**Fig 2:**
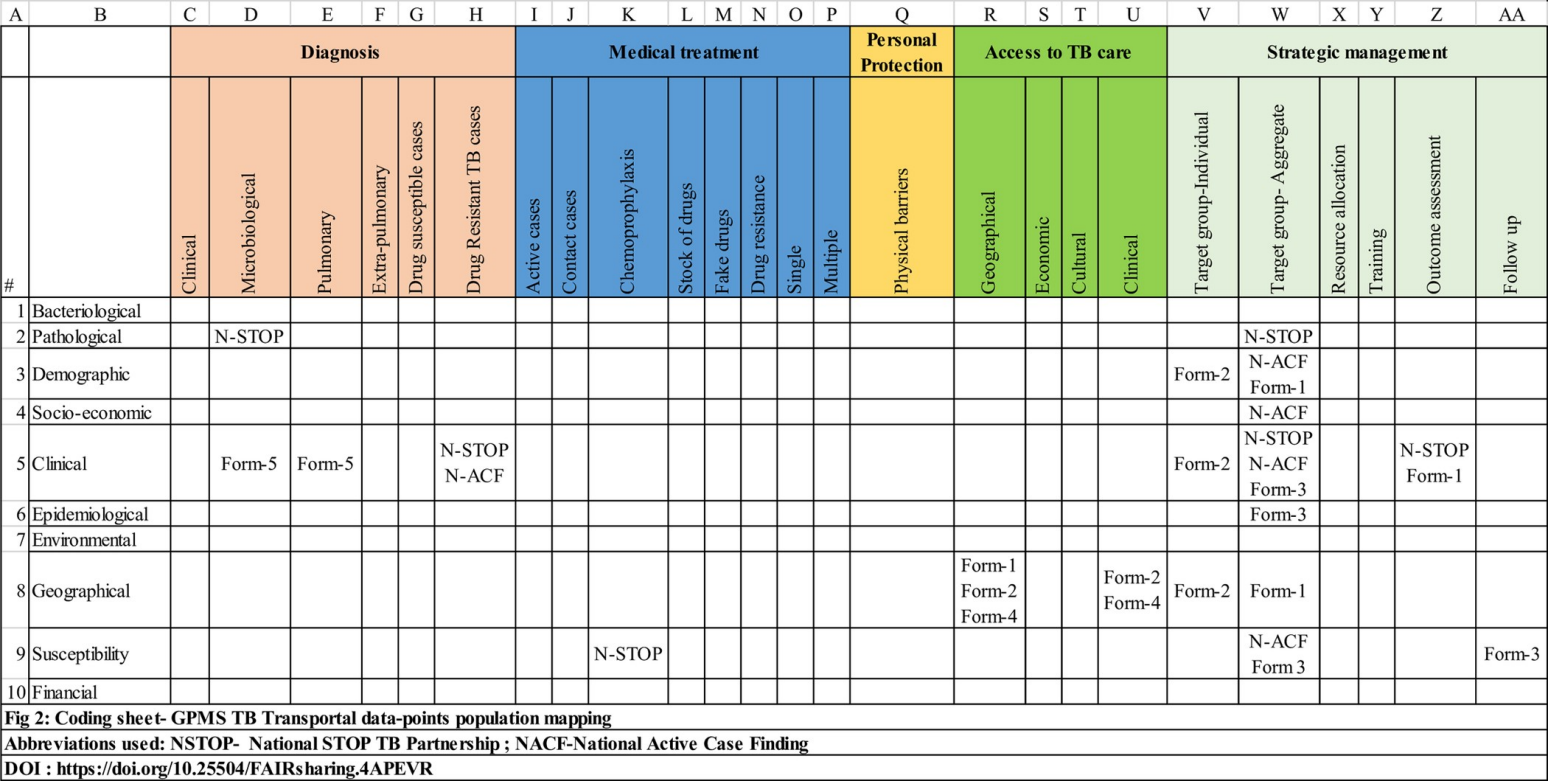
Coding sheet- GPMS TB Transportal data-points population mapping.

We examine whether the ontology captures the existing functions of the GPMS TB Transportal and provides pathways to improve its existing capabilities. For validation, the data elements in the GPMS TB Transportal and their source have been mapped onto a Data x TB Management cross-tabulation table (Fig 2) derived from the ontology. The ‘Data’ elements represent the rows and the ‘TB Management’ elements the columns. A null entry indicates the absence of the corresponding combination of data elements in the Transportal; a double entry indicates the integration of the corresponding combination of data elements from two sources. Only the meta-data is used for mapping and not the data values.

The coding is binary–whether a source provides data corresponding to a cell (Data x TB Management element combination) or not (Fig 2). The unit of coding is the data element in the GPMS TB Transportal. The source name abbreviation is used to encode an entry. The coding is by consensus among the three authors of the paper. Multiple data elements from the same source mapped to a cell are coded only once and are not weighted by the number of elements. Overlapping data elements from different sources are encoded separately. Overall, the cross-tabulation helps visualize: (a) the data available, (b) the data unavailable, and (c) the data sources for TB management in the Transportal.

## Results and inference

The mapping of the platform populated a critical set of GPMS pathways for some of the crucial areas of programmatic concern for TB in the country (S1 Table).

Under Form 1 (W3, W8), GPMS identifies District, Primary health center, Sub-center, Tuberculosis Unit, and Designated Microscopy Center (DMC) for the individual/case identified. This helps identify the relevant risk groups or epidemiologically sensitive areas for early identification of presumptive TB cases. In this case, GPMS is collecting data as part of the Active Case Finding (ACF) TB pilot project in Karnataka, India [18].

*Form 1*: *{Geographic*, *Demographic data} X {Strategic Management-aggregate}*

Form 2 (V3, V5, V8, U8) records Age, Sex, Address, Cough, Fever, DMC lab and adds details for the case in consideration. It provides individual data and includes particulars of all family members, their medical history, and indications of TB before TB treatment and current access to TB care. Hence, it is used for systematic screening of singular presumptive cases within those relevant risk groups or epidemiologically sensitive areas.

*Form 2*: *{Demographic*, *Geographical*, *clinical data} X {Access to TB Care*, *Strategic Management-individual}*

Form 3 (W5, W6, W9, AA9) includes Number of people tested, Number of children below 6 years, Total number of Diabetes Patients, etc. It is the aggregate of all presumptive cases notified to DMCs within a particularly sensitive area.

*Form 3*: *{Clinical*, *susceptibility*, *epidemiological data} X {Strategic management–aggregate*, *follow-up}*

Form 4 (R8, U8) records data on diagnostic tests, clinical examinations, or other procedures to detect active cases with high accuracy. A primary diagnostic tool like sputum smear microscopy is corroborated with routine high sensitivity Chest X-Ray (CXR) for both sputum negative and sputum positive presumptive patients. CXR lesions or other clinical susceptibility cases undergo CBNAAT tests.

*Form 4*: *{Geographical*, *Clinical data} X {Geographical*, *Clinical Access to TB Care}*

Form 5 (D5, E5) reports the Total number of Patients Undergone X-Ray, Total number of Patient Tested Positive From Total Sputum Samples, and Total number under CBNAAT tests. It reports the individual and aggregated data of detected cases at the district level.

*Form 5*: *{Clinical data} X {Microbiological and Pulmonary diagnosis}*

GPMS TB Transportal through its digital applications accelerates TB case detection and yields additional cases. It enables capturing of demographic and geographic details of individual case-based transmission dynamics in-group settings. The records on presumptive cases are notified in primary health centers or DMCs. It further integrates a combination of clinical queries, radiographic and microbiologic testing for enhanced and intensive case finding connected to a DMC, along with details of testing and test centers. The aggregate list of active cases in targeted settings along with a critical range of data notifies Nikshay, of the additional confirmed TB patients with personal and medical records.

The systematic mapping (Fig 2) based on the ontology also demonstrates the possibilities of developing GPMS into a more robust and efficient system with learning capabilities. There are possible surveillance pathways currently not delivered by GPMS TB Transportal, that are crucial for effective treatment strategies. For instance, supporting the financial data for economic access to TB care (S10) such as beneficiaries of healthcare programs and reimbursements (S10). Cash transfers are provided for nutritional support to all tuberculosis (TB) patients in India. To avail the benefit, a list of active cases notified by Nikshay along with their financial data (S10)—Aadhar or National Identification No, Bank Account No, Bank branch details—are provided to Public Financial Management System (PFMS) that notifies banks to disburse funds. Digital records of TB patients will enhance targeted delivery of benefits. Forms 2–5 of GPMS with integrated data on personal identification such as Sex, Age, Name, Mobile Number, Address, High Risk: Village/Place Name, ration card details, medical history, and treatment records could provide a more complete and accurate beneficiary list of TB patients in a district.

We also identify the case of drug-resistant TB. Clinical data for Drug-resistant TB cases are collected in the ACF database (H5). Regular, comprehensive surveillance for drug-resistant TB helps improved case finding. The management of DR-TB is critical and based on laboratory confirmation of TB and a clear understanding of drug resistance aided by Drug Susceptibility Testing (DST) to ensure accurate diagnosis and early intervention of appropriate treatment.

Clinical and pathological data on microbiological diagnosis (D2, D5) could be combined with data on a clinical diagnosis like CBNAAT—Cartridge Based Nucleic Acid Amplification Test–(W5, U8) and susceptibility data for strategic management follow-up (AA9) to widen the scope of treatment surveillance. Providing clinical, pathological, and geographic data of contact cases who are under medical treatment (J5, J2), and thereby deliver information to better understand the TB transmission dynamics for allocation of manpower and resources needs to be explored. Providing clinical, pathological, and geographic data of contact cases who are under medical treatment (J5, J2), and thereby deliver information to better understand the TB transmission dynamics in real-time is also important.

Several other concatenations are also crucial in designing surveillance strategies. Identifying ongoing TB infection sites is crucial for interrupting transmission in TB-prevalent urban areas. Aggregated epidemiological data from target group locations (W6) could enable the documentation of disease trends in each community and the subsequent targeting of resources to where they are needed most. A further possibility is the identification of TB and drug-resistant TB clusters or “hotspots”(H6, H8) at the local level where ongoing transmission occurs, that is critical information for the management of TB, and allocation of manpower and resources.

## Discussion

India’s end TB strategy under the National Strategic Plan 2017–2025 is a multi-pronged approach incorporating patient-centered care and prevention, and supportive systems. TB burden continues to remain huge [19], which will require effective surveillance systems. The ontology lays out a vision and roadmap of a surveillance and monitoring system that, we argue, will allow extended surveillance strategies for digital notification of TB cases, digital applications for monitoring active TB curative and preventive interventions, and interoperability for TB epidemiological trends.

### Strengthening active case finding

Active case finding is considered crucial in TB management especially for early diagnosis and treatment of high-risk populations and contacts of confirmed cases. ACF helps in identifying the missing cases among the vulnerable population in higher proportions when compared to the identification through passive case finding and is proposed under the TB control program in the country. It also facilitates awareness generation among the target population. Identifying cost-effective ACF methods has been suggested to expand the ambit of TB surveillance especially in low- and middle-income settings [20–22]. The National Strategic Plan to End Tuberculosis in India 2020–2025 under the National Tuberculosis Elimination Program (NTEP) acknowledges the need for targeted active case finding in priority populations under its strategic goal of ‘Detect All’ [23]. Progress has been achieved regarding the case finding in terms of decentralizing the diagnostic facilities; however, challenges persist in terms of achieving operational abilities and resource mobilization.

#### Timing of surveillance functions through digitalization

Our analysis reveals the scope for embedding learning surveillance pathways for programmatic implementation of ACF to expand case detection rates. GPMS Transportal has enabled digitalization and cloud-based data reporting capabilities at the grass root levels. It has made possible undertaking the surveillance functions such as reporting, analysis, and feedback in a real-time manner at this level. It provides early identification of the presumptive cases which are to be followed up with further diagnostic processes. However, most of the recording, reporting, and diagnostic processes conducted at the DMCs and -DTCs levels are yet to be digitalized. Digitalization has the potentials for pushing the timing aspect of the overall surveillance activity from ad-hoc and on-demand to periodic and real-time by allowing access and enabling monitoring and action. It will also allow for surveillance functions such as analysis, interpretation, and feedback to happen at more frequent cycles. The framework illustrates the need for improving the timing of the routine functions towards real-time and achieving capabilities for a predictive undertaking of surveillance functions aspired by the 2020–2025 strategy of reaching the target of 95% of notified patients initiated on treatment [24].

ACF needs to be integrated in real-time with the other case-based notification systems to avoid duplicity and improve accuracy. While the ACF data from the GPMS Transportal is integrated with NIKSHAY there are siloes of data collection undertaken on TB incidents by programs such as the National Tobacco Control Program (NTCP) [25]. This must be integrated into the TB notification system. Utilizing Open-API based digital collaboration as suggested by the 2020–2025 strategy for data sharing across various programs is the way forward. This can also result in better identification of missing samples resulting from operational difficulties in undertaking door-to-door surveillance [20]. Thus, digitalization can improve the timing and functional aspects of the learning surveillance system if required resources are put in place and inter-programmatic collaboration is achieved.

#### Data requirements and stakeholders

Data on drug susceptibility, demographic, and socio-economic factors are collected and reported as part of the periodic ACF at a basic level. However, to reduce the cycle time and improve accuracy, pathological, and bacteriological data collection facilities need to be further decentralized and collected much more extensively at the local level. Capacity building at the PHC levels including X-Ray and NAAT facilities for more sensitive and accurate data is required. Apart from this, to extend the current coverage of target population, active involvement of the stakeholders such as ASHA workers, volunteers, and community actors in functions such as training, mobilizing, and sensitizing activities is required.

### Improvement of Direct Benefit Transfer (DBT)

Providing cash transfers for nutritional support combined with active contact investigations is the mainstay of the current programmatic response to TB treatment and care in India. The NTEP has set a target of 100% coverage of nutritional support to TB patients at the time of notification and subsequently during their treatment under Nikshay Poshan Yojana. However, one of the major issues faced by the public health sector in India is an inefficient utilization of the funds allocated. In the case of DBT, the coverage has been low with substantial delays. The limitation of existing tools for DBT poses challenge including the complexity of processes requiring multiple layers of approval and paper-based documentation which overburdened the staff, bulk processing once-a-month, and technological challenges (poor connectivity and issues related to NIKSHAY and PFMS portals) [26]. Several studies on cash transfers in India in other fields such as food subsidy, pension, etc found that cash transfers suffer from leakages and inaccuracies in beneficiary lists [26], lags in financial inclusion and biometric identification resulting in absence of bank accounts or Aadhar cards for beneficiaries [27] or complex and lengthy procedures in linking beneficiary data [28].

The enhancement of currently available digital health records will greatly increase the efficiency of planning and budgeting for health care services [29]. The intended benefit of combining the cash transfer programme for TB with the Aadhar, the citizen identification system, ensures that the right beneficiaries receive social support services at the right place and time. Several issues crop up that require regular updating of records including no access to banking services, Aadhar card registration in progress, or migrant workers’ temporary residence [28]. Frequent updating and linking of records of Aadhar and bank details are therefore critical. GPMS TB Transportal as a digital application enables the collection, storage, use, and dissemination of patients’ digital health information in the ACF mission. It links the Aadhar Number with demographic and biometric information. A more coordinated system between GPMS, Nikshay, and PFMS will enable periodic and an on-demand recording of key information on all beneficiaries and the benefits received. It would enable more prompt enrolment of beneficiaries (as well as the periodic updating of information on beneficiaries to ensure they remain eligible), and facilitate rapid identification of beneficiaries for programs that are scaling up or trying to create synergies with complementary interventions to enhance their impact. This would facilitate the attainment of outcome indicators of India National Strategic Plan for TB 2017–2025, i.e. 90% of notified TB patients receiving financial support through DBT [24].

### Coordinated TB surveillance

Strengthened information infrastructure for TB laboratories could support both the detection and treatment of drug-resistant TB. Laboratory capacity building efforts rarely consider the development of data and information management capabilities. Laboratory services are microbe- or specimen-focused, in contrast to clinical and population health programs. The sensitivity and specificity profiles of emerging diagnostics may differ from those of existing diagnostics, rendering surveillance trend estimates difficult to interpret. There is a critical need to integrate laboratory information systems with those of clinical and population health programs. There is a critical need as well for information systems to track operational activities focused on drug-susceptible and drug-resistant TB, such as infection control programs and drug supply chains, as well as to support performance improvement programs [30].

### Feedback and response

We demonstrated that the data generated as output results is effective for tracking active TB cases, essential for identifying the areas or population groups that are most affected by the disease, and for targeting resources for maximum impact. GPMS’s real-time tracking of active cases is more out-come based supplementing national data like Nikshay, NSTOP, and Nikshay Poshan Yojana. It converts state and national level data repositories to more context-based registries of TB cases with reduced cycle-times. Expert analysis of the data and its interpretation will impact different combinations of interventions. The impact of the response can be captured by the surveillance system and can inform further iterative changes to the interventions. Through iterative cycles and feedback-loops, the effective surveillance actions can be amplified, while the ineffective ones attenuated [31]. GPMS real-time data on presumptive and active cases during the pilot study in southern India followed up with repeated and periodic records of nutritional and socio-economic status, the proximity of infection will report on the possibility of infection. Hence, the pathways through the iterative cycle may be transformed into an advanced learning model as it facilitates the development of predictive systems for TB involving repeated updates on the initial conditions based on the new epidemiological data, and the inference method that naturally lends itself to this purpose, given its time-sequential application.

The learning from the feedback helps redesign the strategies for ending TB as it significantly reduces inaccurate reporting, and ill-informed treatment administration, especially in areas with variable TB transmission patterns. The learning system will help enhance the predictability of TB occurrence and its transmission. It will generate signals of antecedent conditions of TB emergence. These data could help alert public health officials to indicators of elevated TB risk, thereby triggering targeted active surveillance and interventions. The feedback-based system has the potential to be an integral tool for health officials, analysts, and decision-makers. Thus, the ontology supports the rapidly evolving nature of public health surveillance informatics using tools that can be used to establish a communication protocol, describe information flows within a complex setting of TB management, facilitate analysis, and design of the system.

### Way forward

We illustrated the state of the current and potentials for future directions of the surveillance pathways for TB management. We identified the immediate scope for the improvements in the areas of active case finding, managing drug-resistant TB cases, and optimizing financial allocations in-patient care services. In these respects, it is found that digitalization has the potential to improve upon the existing data collection processes in place. The country should tap its advantages in information and mobile technology penetration to implement standardized, interoperable, secure, and transparent systems for information flow. This entails establishing data sharing standards, utilization of open API solutions for inter-entity data exchange for evidence-based decision systems [32]. It would also require moving towards unique health identifiers implemented as part of the universal health care to tackle universally identified issues of duplication and transparency.

On the other hand, the country surveillance system also needs to expand its ambit of data collection and utilization in the context of emerging technologies. The scope of data collection needs to expand to such that of endorsed rapid molecular technologies as suggested by WHO that allows testing of TB resistance to a larger extend at the country level [33]. State-of-the-art pathogenomics has to be incorporated into surveillance systems going forward. However, addressing and ensuring of factors such as community trust, putting in place transparency and accountability mechanisms will be crucial [34].

The ontology for TB surveillance is logical in its deconstruction of the domain, and parsimonious yet complete in the representation of the domain. It can be adapted to future developments in the domain and other contexts. The overall systematic and systemic progress in building up the learning surveillance system would be to establish links horizontally and shift the system vertically down, and it can be further made useful for different stakeholders. This will enable a more effective TB treatment regimen and strengthen health informatics for a more responsive health system.

## Supporting information

S1 Glossary (PDF)

S1 Table Data mapping for validation of ontology of learning surveillance system for TB. (XLSX)
