# Supplementary material for: Envisioning a learning surveillance system for tuberculosis
Source: PLoS One. 2020 Dec 14;15(12):e0243610. doi: 10.1371/journal.pone.0243610 (PMC7735594; doi:10.1371/journal.pone.0243610)
Supplement: S1 Glossary — (PDF) [file pone.0243610.s001.pdf]

S1: Glossary

- **Timing: The timing of surveillance**
- **Ad hoc:** Unplanned surveillance
- **Post hoc:** After reporting surveillance
- **On-demand:** On reporting surveillance
- **Periodic:** Regular interval surveillance
- **Real-time:** At the same time surveillance
- **Predictive:** Forecast surveillance

- **Surveillance: Type of surveillance**
- **Identification:** Locating cases
- **Detection:** Confirming cases
- **Collection:** Collecting samples
- **Analysis:** Studying the cases
- **Interpretation:** Drawing inferences
- **Application:** Applying remedies
- **Reporting:** Providing results
- **Feedback:** Input on results to modify action

- **Data: Type of data**
- **Bacteriological:** Data of Tb bacilli characteristics
- **Pathological:** Data of pathological processes underlying bacterial infection
- **Demographic:** Data of population characteristics
- **Socio-economic:** Data of social and economic determinants
- **Clinical:** Data on health status of patients
- **Epidemiological:** Data of occurrence of diseases in different groups of people
- **Environmental:** Data of environmental characteristics
- **Geographical:** Data on geographic locations
- **Susceptibility:** Data on susceptibility and resistance of Tb bacilli
- **Financial:** Data on financial resources

- **Tuberculosis Management: Diagnosis, managing symptoms, causes, treatment, and prevention of TB**
- **Diagnosis:** Type of diagnosis, Place of diagnosis, delay in diagnosis
- **Medical treatment:** Detection, diagnosis, and treatment with medicines
- **Drug susceptible cases:** Susceptible to first line drugs
- **Drug resistant TB cases:** Resistance to First line, MDR TB, XDR TB
- **Contact cases:** Infected cases with no symptoms or manifestation of disease
- **Chemoprophylaxis:** Treatment given, or action taken to prevent disease in susceptible
- **Stock of drugs:** Supply of drugs in storage and distributional channels
- **Fake drugs:** Counterfeit medicines
- **Drug resistance:** Reduction in effectiveness of a medication
- **Personal Protection:** Personal protective measures

- **Physical barriers:** Items fitted as barriers to entry
- **Access to TB care:** Accessibility for TB care
- **Geographical Access:** Geographical Access to TB care
- **Economic Access:** Economic Access to TB care
- **Cultural Access:** Cultural access to TB care
- **Clinical Access:** Clinical access to TB care
- **Strategic management:** Long-term planning
- **Resource allocation:** Inputs for strategic management
- **Training:** Capacity building and awareness programmes
- **Outcome assessment:** Evaluation of desired outcomes

- **Stakeholders: Those affecting and affected by TB**
- **Health Care Provider:** Professionals in public or private healthcare systems
- **People:** Personnel engaged in delivering the health care services
- **Physician:** Medical practitioner
- **Nurse:** Medical professional providing and monitoring patient care
- **Pharmacist:** Health professionals who dispense prescription medications to patients
- **ASHA worker:** Accredited Social Health Activist (ASHA) a trained community health activist
- **Volunteer:** Medical student interns
- **Treatment supporter:** Persons guiding and motivating TB patients during treatment
- **Entity:** Units involved in healthcare systems
- **Hospital:** An institution providing medical, surgical, and nursing treatment
- **Clinic:** An establishment or hospital department that provides medical care to a condition
- **Pharmacy:** A shop or hospital dispensary where medicinal drugs are prepared or sold.
- **Home:** Place of residence
- **Designated microscopic center:** Quality TB diagnostics centers
- **Tuberculosis unit**: A place where TB patient has been received for treatment
- **Intermediate reference lab (IRL):** TB testing lab
- **Citizen:** User or prospective user of healthcare service in a country
- **Individual:** A person
- **Family:** A group of related people
- **Community:** A social unit with commonality
- **Agency:** An agency is a business, firm, or organization that provides a specific service
- **Public:** Government owned health organizations
- **Private:** Commercial-based health organizations
- **NGO:** Non-for-profit health organizations
- **Academia:** Universities, Research Institutes
